# Comparison of Cognitive Functions in Patients with Autogenous and Reactive Obsessive–Compulsive Disorder

**DOI:** 10.3390/brainsci16090922

**Published:** 2026-08-29

**Authors:** Hatice Ayça Kaloğlu, Mehmet Ünler, Buket Koparal, Çisem Utku, Nevzat Yüksel

**Affiliations:** 1 Department of Psychiatry, Ankara Etlik City Hospital, Ankara 06170, Turkey; 2 Department of Psychiatry, Hatem Hospital, Gaziantep 27010, Turkey; 3 Department of Psychiatry, Gazi University, Ankara 06500, Turkey

**Keywords:** obsessive–compulsive disorder, autogenous obsessions, reactive obsessions, neurocognitive functioning, executive functioning, verbal memory, cognitive flexibility, depression

## Abstract

**Introduction:** Obsessive–compulsive disorder (OCD) is clinically heterogeneous, and the neurocognitive characteristics of autogenous and reactive obsessional presentations remain insufficiently understood. This study compared neurocognitive performance and exploratory clinical–cognitive associations in strictly classified autogenous and reactive OCD presentations. **Methods:** This cross-sectional study included 67 outpatients with OCD classified as having reactive (*n* = 35) or autogenous (*n* = 32) presentations based on clinical evaluation and the Yale–Brown Obsessive Compulsive Scale Symptom Checklist. Patients with mixed presentations were excluded. Executive functioning, attentional control, cognitive flexibility, and verbal learning and memory were assessed using the Wisconsin Card Sorting Test, Stroop Test, and Rey Auditory Verbal Learning Test. Depressive symptoms were assessed using the Beck Depression Inventory. **Results:** The groups were comparable in age, age at OCD onset, illness duration, and overall OCD symptom severity, whereas depressive symptom scores were higher in the autogenous group. Neuropsychological performance was broadly comparable between groups. The autogenous group showed better RAVLT-6 performance, reflecting recall after interference, with a nominally significant unadjusted group difference. This difference remained significant in an exploratory ANCOVA adjusting for depressive symptoms, education, and age at OCD onset, although it did not survive false-discovery-rate correction. Exploratory within-group correlations were observed; however, formal Fisher’s r-to-z comparisons revealed no significant differences between groups. **Conclusions:** Strictly classified autogenous and reactive OCD presentations showed broadly similar neuropsychological profiles, with a preliminary group difference in recall after interference. Within-group correlations should be interpreted as exploratory and hypothesis-generating rather than as evidence of presentation-specific clinical–cognitive mechanisms. Replication in larger samples is warranted.

## 1. Introduction

Obsessive–compulsive disorder (OCD) is a chronic and debilitating psychiatric condition characterized by recurrent obsessions and/or compulsions that cause significant distress and functional impairment [1]. Although OCD is classified as a single diagnostic entity, it is widely recognized as a highly heterogeneous disorder, showing substantial variability in symptom dimensions, clinical course, age at onset, comorbidity patterns, treatment response, and functional outcomes [2]. This heterogeneity is increasingly considered a major challenge in elucidating the neurobiological and cognitive mechanisms underlying the disorder and in interpreting the inconsistent findings reported across neuropsychological studies [2,3].

Over the past several decades, extensive research has examined cognitive functioning in individuals with OCD. Challenges have been reported across multiple domains, including executive functioning, inhibitory control, attention, memory, cognitive flexibility, planning, and decision-making [3,4]. Nevertheless, the magnitude and consistency of these disorders remain a matter of ongoing debate. Meta-analytic evidence indicates that, although patients with OCD often perform worse than healthy controls on neuropsychological measures, the observed effect sizes are generally small to moderate and show considerable variability across studies [3,4]. These inconsistencies have led researchers to question whether cognitive dysfunction represents a core feature of OCD or whether it is shaped by clinical and methodological factors. Recent reviews have emphasized that variables such as symptom severity, illness duration, depressive symptom burden, medication status, motivational factors, metacognitive processes, and the ecological validity of neuropsychological measures may substantially contribute to the heterogeneity of cognitive findings in OCD [5].

In this context, the investigation of clinically meaningful OCD symptom profiles has emerged as a promising approach for clarifying the sources of variability in cognitive performance. This perspective assumes that cognitive dysfunction may not be uniformly distributed across all individuals with OCD but may instead be linked to specific symptom profiles or underlying psychopathological mechanisms. Support for this view comes from evidence suggesting that some cognitive abnormalities observed in OCD, including challenges reported in unaffected relatives, may reflect trait-related vulnerability markers rather than solely state-dependent consequences of the disorder [6]. Similarly, findings from decision-making research indicate that individuals with OCD tend to perform less advantageously on tasks involving uncertainty and risk, suggesting alterations in cognitive processes related to evaluation and response selection [7]. However, the marked clinical heterogeneity within OCD populations complicates the identification of symptom-specific cognitive mechanisms and may partially account for the inconsistencies reported in the literature.

One of the most influential models proposed to conceptualize OCD heterogeneity is the autogenous–reactive obsession model introduced by Lee and Kwon [8]. According to this framework, obsessions can be broadly classified into two qualitatively distinct categories. Autogenous obsessions are characterized by their spontaneous and internally generated nature, often emerging abruptly in the absence of identifiable external triggers. These obsessions typically involve themes related to aggression, sexuality, or religion and are experienced as highly ego-dystonic, intrusive, and distressing. In contrast, reactive obsessions are generally elicited by identifiable environmental stimuli and are commonly associated with contamination, doubt, checking, symmetry, or ordering concerns. Compared with autogenous obsessions, reactive obsessions are more closely linked to externally triggered threat-related concerns and preventive behavioral responses [8,9].

Importantly, the autogenous–reactive distinction extends beyond differences in symptom content and encompasses broader cognitive and emotional processes. Individuals with autogenous obsessions have been shown to exhibit greater tendencies toward thought–action fusion, inflated responsibility, guilt, and maladaptive interpretations of intrusive thoughts. In contrast, reactive obsessions appear to be more strongly associated with attentional monitoring, threat detection, uncertainty management, and behavioral control strategies [8,9]. Recent systematic evidence further underscores that distinct metacognitive belief profiles are differentially distributed across OCD symptom dimensions, with repugnant/autogenous intrusions being notably linked to beliefs regarding the need to control thoughts and the dangerousness of thoughts, whereas reactive dimensions correlate more strongly with beliefs concerning cognitive competence and threat estimation [10]. Although these appraisal-related and metacognitive processes are conceptually distinct from performance-based neuropsychological functions, they may influence attentional allocation, interference processing, cognitive control, and strategic learning. Given these differences in cognitive appraisal, metacognition, and emotional processing, it is plausible that autogenous and reactive obsessional presentations may be associated with distinct neuropsychological profiles. Therefore, the autogenous–reactive model provides a theoretically meaningful framework for examining potential cognitive heterogeneity within OCD.

Clinical investigations have further suggested that patients with autogenous and reactive obsessional presentations may differ in associated psychopathological features. In particular, individuals with autogenous obsessions have been reported to experience higher levels of depressive symptoms and emotional distress, even when overall OCD severity is comparable between groups [11]. This observation is particularly relevant to neuropsychological research, as depressive symptomatology has been consistently associated with impairments in attention, executive functioning, and memory. Consequently, depressive symptom burden represents an important potential confounder in cognitive performance and should be carefully considered when evaluating differences between autogenous and reactive obsessional presentations [5,11].

Despite the theoretical relevance of the autogenous–reactive distinction, empirical studies directly comparing the cognitive functioning of these two presentations remain relatively scarce. Existing findings have generally failed to demonstrate robust or widespread neuropsychological differences between the groups. For example, Aydın and colleagues compared patients with autogenous and reactive obsessional profiles on measures of executive functioning, cognitive flexibility, attention, and verbal memory using the WCST, Stroop Test, and RAVLT, reporting largely comparable performance across groups [12]. Collectively, these findings suggest that if cognitive differences between these presentations exist, they are likely to be subtle and domain-specific rather than indicative of generalized cognitive impairment. Furthermore, methodological factors such as sample size, depressive symptom burden, and variations in neuropsychological assessment strategies may have contributed to the variability of reported results [5,11,12,13].

A major methodological challenge in this area of research is the frequent overlap between autogenous and reactive obsessional symptoms within individual patients. Such symptom overlap may obscure potential symptom profile-specific cognitive differences and may partially explain the limited differentiation reported in previous studies. In the present study, patients were classified into strictly defined “pure” autogenous or “pure” reactive obsessional groups based on the Y-BOCS Symptom Checklist, using predefined classification criteria. By minimizing symptom overlap between the two groups, this design was intended to allow for a more precise evaluation of whether distinct obsessional profiles are associated with differences in neurocognitive functioning.

Given the theoretical differences in appraisal processes, threat monitoring, and internally versus externally triggered intrusive experiences, we hypothesized that autogenous and reactive obsessional presentations would show differential neurocognitive profiles. Specifically, based on the heightened environmental threat monitoring and behavioral control demands typical of reactive obsessions, we tentatively anticipated that reactive presentations might demonstrate greater relative difficulties in executive flexibility (WCST) and interference control (Stroop Test). Conversely, because autogenous obsessions are characterized by spontaneous, internally generated intrusive themes and higher emotional distress, we hypothesized that verbal memory and learning processes under interference (RAVLT) might show distinct vulnerability patterns, although a bidirectional possibility was maintained given the scarcity and inconsistency of the previous literature. Because prior empirical comparisons directly contrasting strictly defined presentations are limited, all analyses of clinical–cognitive associations were considered exploratory.

## 2. Materials and Methods

### 2.1. Study Design and Participants

This cross-sectional study was conducted at the Psychiatry Outpatient Clinic of Gazi University Faculty of Medicine between March 2017 and May 2017. A total of 81 consecutive outpatients with obsessive–compulsive disorder (OCD) were screened. Eligible participants were 18–65 years of age, had completed at least five years of formal education, met the Diagnostic and Statistical Manual of Mental Disorders, Fifth Edition (DSM-5) diagnostic criteria for OCD [1], and had a total Yale–Brown Obsessive Compulsive Scale (Y-BOCS) score of ≥8, indicating at least mild symptom severity.

Psychiatric diagnoses and psychiatric comorbidity profiles were evaluated by an experienced board-certified psychiatrist using the Structured Clinical Interview for DSM-5 (SCID-5) [14]. Patients with active major depressive disorder, psychotic disorders, bipolar disorder, or substance use disorders were excluded. Participants were strictly classified into autogenous or reactive presentations using a predefined operational rule based on the Y-BOCS Symptom Checklist: the autogenous group comprised patients presenting exclusively with aggressive/harm-related, sexual, or religious obsessions, whereas the reactive group comprised patients presenting exclusively with contamination/cleaning, symmetry/ordering, or doubt/checking obsessions. Out of the 81 screened patients, 14 presenting with mixed obsessional dimensions (i.e., endorsing symptoms from both categories) were excluded, resulting in a final sample of 67 patients (reactive: *n* = 35; autogenous: *n* = 32). Classification was independently verified by a second senior psychiatrist, with full diagnostic concordance (inter-rater agreement: Cohen’ s kappa: 1.00). The neuropsychological battery was administered individually by a trained investigator who was blinded to the subtype classification.

All participants were receiving pharmacological treatment at the time of assessment. Medication information was recorded categorically, including the use of selective serotonin reuptake inhibitors (SSRIs), serotonin–norepinephrine reuptake inhibitors (SNRIs), tricyclic antidepressants (TCAs), and atypical antipsychotic augmentation. Detailed data regarding daily dosage, treatment duration, treatment response, cumulative medication exposure, and anticholinergic burden were not systematically collected.

The exclusion criteria were as follows: (a) a BDI score of ≥17; (b) major neurological, metabolic, or active inflammatory disorders; (c) a history of severe head trauma involving loss of consciousness or previous neurosurgical intervention; (d) alcohol or substance use disorder; (e) current use of medications with substantial central nervous system depressant effects, including benzodiazepines or barbiturates; (f) color blindness or hearing impairment; and (g) the presence of both autogenous and reactive obsessional dimensions in the same individual (i.e., mixed presentations).

Written informed consent was obtained from all participants after they received a complete explanation of the study procedures. The study protocol was approved by the Gazi University Ethics Commission (Approval Decision No. 34) and was conducted in accordance with the Declaration of Helsinki.

### 2.2. Psychometric and Neuropsychological Assessment

Sociodemographic Data Form: A sociodemographic data form developed by the researchers was used to collect information on participants’ age, sex, educational level, marital status, employment status, age at OCD onset, illness duration, treatment history, and other clinical characteristics.

Yale–Brown Obsessive Compulsive Scale (Y-BOCS): The Yale–Brown Obsessive Compulsive Scale (Y-BOCS) is a clinician-rated instrument used to assess the severity of obsessive–compulsive symptoms [15]. The Turkish version has demonstrated satisfactory validity and reliability [16].

Beck Depression Inventory (BDI): The Beck Depression Inventory (BDI) is a 21-item self-report instrument used to assess the severity of depressive symptoms [17]. The Turkish version has been shown to be valid and reliable in clinical populations [18].

Wisconsin Card Sorting Test (WCST): The Wisconsin Card Sorting Test (WCST) is a widely used measure of executive functioning that assesses cognitive flexibility, abstract reasoning, set shifting, and problem-solving abilities [19]. In the present study, the WCST was administered and scored according to the Turkish standardization study conducted by Karakaş [20]. Although multiple indices can be derived from the test, the primary analyses focused on Categories Completed (CC), Perseverative Errors (PE), and Conceptual-Level Responses (CLR). These indices were selected because they reflect key components of executive functioning, including concept formation, cognitive flexibility, and set shifting [21,22].

Stroop Test (ST): The Stroop Test assesses selective attention, cognitive control, response inhibition, and resistance to interference [23]. In the present study, the TÜBİTAK Basic Sciences Research Group (TBAG) version was administered [20]. Completion times, numbers of errors, and self-corrections were recorded for each condition. Completion time on the fifth condition, reflecting interference performance, was used as the primary indicator of inhibitory control.

Rey Auditory Verbal Learning Test (RAVLT): The Rey Auditory Verbal Learning Test (RAVLT) evaluates verbal learning and memory, including immediate recall, learning capacity, recall after interference, delayed recall, and recognition memory [24]. Based on the Turkish standardization study, the following RAVLT indices were included in the analyses: RAVLT-1, reflecting initial recall; RAVLT-5, reflecting verbal learning performance; RAVLT-6, reflecting recall after interference; RAVLT-7, reflecting short-delay recall; RAVLT-8, reflecting long-delay recall; and recognition performance.

### 2.3. Statistical Analysis

Statistical analyses were performed using IBM SPSS Statistics for Windows, Version 26.0 (IBM Corp., Armonk, NY, USA). Descriptive statistics are presented as means and standard deviations (SDs) for continuous variables and as frequencies and percentages for categorical variables. The normality of data distribution was tested using the Shapiro–Wilk and Kolmogorov–Smirnov tests, supplemented by visual inspection of histograms and Q-Q plots. Continuous variables showing acceptable normality were compared using Student’s independent-samples t-test. For variables demonstrating mild skewness (e.g., illness duration and WCST perseverative errors), non-parametric Mann–Whitney U tests were also conducted, which confirmed identical patterns of statistical significance. Categorical variables were examined using Pearson’s chi-square test or Fisher’s exact test where appropriate. Associations between clinical variables and neuropsychological scores were evaluated using Pearson’s correlation coefficients. Effect sizes for continuous variables were computed as Hedges’ g with approximate 95% confidence intervals. To address potential Type I error inflation across the ten neuropsychological comparisons in Table 1, *p*-values were adjusted using the Benjamini–Hochberg false discovery rate (FDR) procedure (pFDR). In addition, an analysis of covariance (ANCOVA) was conducted for RAVLT-6, covarying for BDI score, years of education, and age at OCD onset. Differences between independent correlation coefficients across the two groups were formally evaluated using two-tailed Fisher’s r-to-z tests. Findings surviving multiple-testing correction or multivariable adjustment were considered statistically significant, whereas unadjusted *p*-values < 0.05 were reported as nominally significant. Statistical significance was set at *p* < 0.05. An a priori sample size calculation performed using G*Power software (version 3.1) indicated that a minimum total sample size of 60 participants (30 per group) was required to detect a medium-to-large effect size (Cohen’s d = 0.70) with a statistical power of 0.80 and a two-tailed alpha of 0.05. The final recruited sample of 67 patients (reactive: *n* = 35; autogenous: *n* = 32) fully met and exceeded this target.

## 3. Results

A total of 67 patients with obsessive–compulsive disorder (OCD) were included in the study. Based on the Yale–Brown Obsessive Compulsive Scale Symptom Checklist (Y-BOCS-SC), participants were classified into two groups: a reactive OCD group (*n* = 35) and an autogenous OCD group (*n* = 32). The reactive OCD group included patients with contamination, symmetry/ordering, and doubt/checking obsessions, whereas the autogenous OCD group included patients with aggressive/harm-related, religious, and sexual obsessions. The sociodemographic and clinical characteristics of the reactive and autogenous OCD groups are presented in Table 2. No significant between-group differences were observed in age, age at OCD onset, illness duration, or OCD symptom severity. However, BDI scores differed significantly between groups. Patients in the autogenous OCD group had higher BDI scores (*M* = 9.38, *SD* = 4.19) than those in the reactive OCD group (*M* = 5.74, *SD* = 3.62; *t* = −3.779, *p* < 0.001).

All participants were receiving pharmacological treatment at the time of assessment. Medication categories included selective serotonin reuptake inhibitors (SSRIs), serotonin–norepinephrine reuptake inhibitors (SNRIs), tricyclic antidepressants (TCAs), and atypical antipsychotic augmentation. SSRI use was reported in 54.3% (*n* = 19) of the reactive OCD group and 62.5% (*n* = 20) of the autogenous OCD group. SNRI use was present in 2.9% (*n* = 1) of the reactive group and 3.1% (*n* = 1) of the autogenous group, whereas TCA use was present in 8.6% (*n* = 3) of the reactive group and 3.1% (*n* = 1) of the autogenous group. Atypical antipsychotic augmentation was administered to 28.6% (*n* = 10) of the reactive group and 25.0% (*n* = 8) of the autogenous group. As detailed in Table 2, comparative analyses revealed no statistically significant between-group differences across any of the recorded medication categories (*p* > 0.05).

Comparative neuropsychological test results for the reactive and autogenous OCD groups, along with effect sizes (Hedges’ g), 95% confidence intervals, and FDR-adjusted *p*-values, are presented in Table 1. A nominally significant unadjusted between-group difference was observed only for RAVLT-6, reflecting recall after interference (*t* = −2.098, *p* = 0.040, Hedges’ g = −0.51, 95% CI [−0.99, −0.02]), with patients in the autogenous OCD group recalling more words (*M* = 6.34, *SD* = 2.18) than those in the reactive OCD group (*M* = 5.37, *SD* = 1.59). However, this difference did not survive Benjamini–Hochberg FDR correction across the ten neuropsychological comparisons (pFDR = 0.398). When this comparison was repeated as an exploratory ANCOVA adjusting for BDI score, educational level, and age at OCD onset, the main effect of the group remained statistically significant (*F*(1, 62) = 5.53, *p* = 0.022, partial η^2^ = 0.082), with an adjusted mean advantage of 0.995 words for the autogenous group. No statistically significant or nominally significant differences were observed on any other RAVLT indices, the Stroop Test, or the Wisconsin Card Sorting Test (WCST).

**Table 1:** Between-group comparisons of neuropsychological measures with effect sizes and FDR-adjusted *p*-values.

Measure	Reactive M ± SD	Autogenous M ± SD	*t*	*p* (Unadjusted)	Hedges’ g	95% CI for g	*p* (FDR)
RAVLT-1 (initial recall)	5.80 ± 1.43 (*n* = 35)	6.12 ± 1.96 (*n* = 32)	−0.779	0.439	−0.188	−0.669 to 0.292	0.901
RAVLT-5 (verbal learning)	11.66 ± 2.20 (*n* = 35)	12.06 ± 2.17 (*n* = 32)	−0.759	0.451	−0.184	−0.664 to 0.297	0.901
RAVLT-6 (recall after interference)	5.37 ± 1.59 (*n* = 35)	6.34 ± 2.18 (*n* = 32)	−2.098	0.040	−0.507	−0.994 to −0.020	0.398
RAVLT-7 (short-delay recall)	9.63 ± 2.88 (*n* = 35)	10.00 ± 2.58 (*n* = 32)	−0.554	0.581	−0.134	−0.614 to 0.346	0.960
RAVLT-8 (long-delay recall)	9.06 ± 2.51 *(n* = 35)	9.34 ± 3.00 (*n* = 32)	−0.425	0.672	−0.103	−0.583 to 0.377	0.960
RAVLT recognition	12.89 ± 2.22 (*n* = 35)	13.31 ± 1.94 (*n* = 32)	−0.834	0.407	−0.202	−0.682 to 0.279	0.901
Stroop fifth condition (s)	27.46 ± 10.60 (*n* = 35)	27.60 ± 9.72 (*n* = 32)	−0.057	0.954	−0.014	−0.493 to 0.466	0.987
WCST categories completed	4.69 ± 1.49 (*n* = 35)	4.97 ± 1.56 (*n* = 32)	−0.760	0.450	−0.184	−0.664 to 0.296	0.901
WCST perseverative errors	17.41 ± 11.41 (*n* = 35)	17.47 ± 11.83 (*n* = 32)	−0.019	0.985	−0.005	−0.496 to 0.486	0.987
WCST conceptual-level responses	60.80 ± 12.80 (*n* = 35)	60.75 ± 12.75 (*n* = 32)	0.016	0.987	0.004	−0.476 to 0.483	0.987

Note: Values are means ± standard deviations. Negative effect sizes indicate lower values in the reactive group than in the autogenous group. Hedges’ g is based on reactive minus autogenous group means, with approximate 95% confidence intervals. The Benjamini–Hochberg procedure was applied across the ten neuropsychological comparisons. RAVLT-6 was nominally significant before correction but did not remain significant after FDR correction.

**Table 2:** Sociodemographic, clinical, and medication characteristics of the reactive and autogenous OCD groups.

Variable	Reactive OCD (*n* = 35)	Autogenous OCD (*n* = 32)	Test Statistic	*p*-Value
Age, years	29.97 ± 9.00	30.65 ± 10.15	*t* = −0.291	0.772
Sex, female/male, *n* (%)	16 (45.7)/19 (54.3)	17 (53.1)/15 (46.9)	χ^2^ = 0.367	0.544
Education status, *n* (%)			χ^2^ = 0.027; df = 2	0.987
Primary/middle school	5 (14.3)	5 (15.6)		
High school	12 (34.3)	11 (34.4)		
Associate degree/university	18 (51.4)	16 (50.0)		
OCD duration, months	93.26 ± 76.34	124.00 ± 93.26	*t* = −1.468	0.147
Age at OCD onset, years	22.46 ± 8.52	20.44 ± 7.80	*t* = 1.012	0.315
Y-BOCS total score	19.34 ± 5.65	20.66 ± 6.78	*t* = −0.858	0.395
BDI score	5.74 ± 3.62	9.38 ± 4.19	*t* = −3.779	<0.001
SSRI use, *n* (%)	19 (54.3)	20 (62.5)	χ^2^ = 0.464	0.496
SNRI use, *n* (%)	1 (2.9)	1 (3.1)	Fisher’s exact	1.000
TCA use, *n* (%)	3 (8.6)	1 (3.1)	Fisher’s exact	0.615
Atypical antipsychotic augmentation, *n* (%)	10 (28.6)	8 (25.0)	χ^2^ = 0.109	0.742

Values are presented as mean ± SD unless otherwise indicated. Independent-sample Student’s *t* test was used for continuous variables, and Pearson’s chi-square test was used for categorical variables, except when Fisher’s exact test was used because of small expected cell counts. For the education-status comparison, primary and middle school categories were combined because of small cell counts. OCD, obsessive–compulsive disorder; Y-BOCS, Yale–Brown Obsessive Compulsive Scale; BDI, Beck Depression Inventory; SSRI, selective serotonin reuptake inhibitor; SNRI, serotonin–norepinephrine reuptake inhibitor; TCA, tricyclic antidepressant; SD, standard deviation.

Table 3 presents Pearson correlation analyses examining the associations of clinical variables—including OCD symptom severity, as assessed using the Yale–Brown Obsessive Compulsive Scale (Y-BOCS); depressive symptom severity (BDI scores); illness duration; and age at OCD onset—with neuropsychological test performance in the reactive OCD group. Neuropsychological measures included verbal learning and memory performance (RAVLT), attentional control and interference performance (Stroop Test), and executive functioning (WCST).

In the reactive OCD group, Y-BOCS scores were positively correlated with completion time on the fifth Stroop condition, reflecting interference performance (*r* = 0.469, *p* < 0.01). In addition, Y-BOCS scores were negatively correlated with WCST Categories Completed (*r* = −0.382, *p* < 0.05) and positively correlated with WCST Perseverative Errors (*r* = 0.417, *p* < 0.05). Illness duration was positively correlated with completion time on the fifth Stroop condition (*r* = 0.403, *p* < 0.05) and negatively correlated with both WCST Categories Completed (*r* = −0.554, *p* < 0.01) and WCST Conceptual Level Responses (*r* = −0.334, *p* < 0.05). BDI scores were positively correlated with WCST Perseverative Errors (*r* = 0.475, *p* < 0.01). Furthermore, a higher age at OCD onset was associated with lower scores on RAVLT-1 (*r* = −0.350, *p* < 0.05) and RAVLT-6 (*r* = −0.394, *p* < 0.05), longer completion time on the fifth Stroop condition (*r* = 0.373, *p* < 0.05), and a greater number of WCST Perseverative Errors (*r* = 0.403, *p* < 0.05).

Table 4 summarizes Pearson correlation analyses for the autogenous OCD group. In this group, age at OCD onset was negatively correlated with several measures of verbal learning and memory performance, including RAVLT-1 (*r* = −0.408, *p* < 0.05), RAVLT-5 (*r* = −0.391, *p* < 0.05), RAVLT-6 (*r* = −0.436, *p* < 0.05), RAVLT-7 (*r* = −0.430, *p* < 0.05), RAVLT-8 (*r* = −0.546, *p* < 0.01), and recognition performance (*r* = −0.413, *p* < 0.05). Additionally, age at OCD onset was positively correlated with completion time on the fifth Stroop condition (*r* = 0.596, *p* < 0.01). Y-BOCS scores were negatively correlated with RAVLT-8 long-delay recall (*r* = −0.351, *p* < 0.05) and positively correlated with completion time on the fifth Stroop condition (*r* = 0.362, *p* < 0.05). BDI scores were negatively correlated with RAVLT-6 (*r* = −0.364, *p* < 0.05) and RAVLT-7 (*r* = −0.406, *p* < 0.05). Illness duration was negatively correlated with WCST Conceptual Level Responses (*r* = −0.370, *p* < 0.05). In the autogenous OCD group, no significant correlations were observed between WCST Categories Completed or Perseverative Errors and any clinical variables. Finally, Y-BOCS and BDI scores showed a large positive correlation (*r* = 0.636, *p* < 0.01).

Formal between-group comparisons of correlation coefficients using two-tailed Fisher’s r-to-z tests revealed no statistically significant differences in correlation magnitude across reactive and autogenous groups for any of the clinical–cognitive associations examined (all *p* > 0.05; Supplementary Table S1).

## 4. Discussion

The present study found largely comparable neuropsychological performance between strictly defined autogenous and reactive OCD groups, with the exception of better performance on the RAVLT-6 interference list in the autogenous group. However, the observed within-group associations provide only preliminary indications of potentially relevant clinical–cognitive relationships. Because the subgroup samples were small and the correlation analyses involved multiple uncorrected comparisons, these findings should not be interpreted as evidence of subtype-specific cognitive differences or distinct underlying mechanisms.

More specifically, the lack of statistically significant between-group differences in Stroop Test performance and WCST indices should be framed in terms of statistical precision and power rather than definitive proof of an absence of effect. Although these findings suggest that the autogenous–reactive distinction may not manifest as large divergent profiles in executive flexibility or response inhibition—consistent with the observations of Aydın et al. [12]—the modest sample size limits the precision to detect small-to-subtle effect sizes. The absence of marked differentiation, even within strictly defined pure groups, raises the possibility that certain neurocognitive difficulties in OCD represent cross-cutting dimensional features rather than phenotype-specific impairments. Furthermore, although the autogenous–reactive model has been useful in explaining differences in symptom phenomenology, threat appraisal, and metacognitive interpretations, performance-based neuropsychological measures may be less sensitive to these qualitative dimensions of obsessions. Previous evidence also indicates that executive-function findings in OCD may vary according to the specific task used, the cognitive index examined, and clinical or methodological factors such as symptom severity, medication status, and affective symptoms [5]. Findings based specifically on Stroop- and WCST-derived measures likewise suggest that inhibitory control and cognitive flexibility may be influenced by clinical factors, including symptom severity and depressive symptoms [25]. Taken together, these considerations suggest that any potential neuropsychological differences between autogenous and reactive OCD may be small and should be interpreted cautiously, particularly because the isolated RAVLT-6 finding did not remain significant after correction for multiple comparisons.

The only nominally significant unadjusted between-group difference was observed for RAVLT-6 scores, with the autogenous group demonstrating better recall after interference. In the standard RAVLT paradigm, RAVLT-6 (Trial 6) reflects the immediate recall of the original word list (List A) following presentation and recall of the interference list (List B). Although this single-trial index was no longer significant following FDR multiplicity correction, the group difference remained statistically robust after controlling for BDI scores, education, and age at OCD onset in an ANCOVA (F(1, 62) = 5.53, *p* = 0.022, partial η^2^ = 0.082), suggesting that the effect is not a simple artifact of depressive symptom burden. Notably, an apparent internal tension emerges when integrating group-level comparisons with within-group associations: the autogenous group exhibited both higher overall depressive symptom severity and superior RAVLT-6 performance compared with the reactive group, yet within the autogenous group, higher BDI scores were negatively correlated with RAVLT-6 recall (r = −0.364). This juxtaposition argues against a simplistic, linear depression-mediated account of the group difference. Although elevated depressive distress may confer vulnerability to interference during memory retrieval [26], the present data cannot determine whether the isolated RAVLT-6 difference reflects depressive symptoms, differences in obsessional phenomenology, unmeasured cognitive strategies, or chance variation. Nevertheless, subclinical depressive symptom burden alone may not fully account for the observed RAVLT-6 difference, particularly because no broader between-group advantage was observed across the remaining verbal learning and memory indices. Accordingly, the isolated RAVLT-6 difference may reflect a preliminary difference in performance under interference-related learning conditions; however, because it did not survive correction for multiple comparisons, it should be interpreted cautiously and requires replication.

Consistent with previous clinical observations, patients in the autogenous group exhibited significantly higher depressive symptom severity than those in the reactive group. Autogenous obsessions—particularly intrusive aggressive, sexual, and religious thoughts—are often experienced as highly ego-dystonic and may be associated with guilt, shame, self-criticism, and maladaptive interpretations of unwanted thoughts. Recent dimensional evidence further indicates that repugnant or taboo obsessional thoughts are more closely associated with depressive symptoms and negative affect than other OCD symptom dimensions [27]. Although individuals with BDI scores of ≥17 were excluded according to the predefined study criterion, the remaining between-group difference indicates that depressive symptom burden remained a relevant potential confounder when interpreting the neuropsychological findings. This issue is particularly important because depressive symptoms, including subthreshold symptoms, have been associated with poorer cognitive control, while depression more broadly may affect attention, processing speed, executive functioning, and memory [28,29]. Moreover, the positive association between Y-BOCS and BDI scores in the autogenous group may indicate a possible relationship between obsessive–compulsive symptom severity and depressive distress in this subgroup; however, this finding was exploratory and does not establish that the association is specific to the autogenous presentation. Given the cross-sectional and correlational nature of these analyses, this association should be interpreted as exploratory and does not establish a directional or causal relationship.

Within the reactive OCD group, greater OCD symptom severity was associated with longer Stroop interference time, fewer Categories Completed (CC) on the WCST, and more Perseverative Errors (PE). In addition, longer illness duration was associated with longer Stroop interference time, fewer CC, and lower Conceptual Level Responses (CLR). Taken together, these nominal within-group associations may reflect a possible relationship between clinical burden and several executive-function measures in the reactive group. They should nevertheless be viewed as preliminary signals rather than as evidence of reduced executive efficiency specific to reactive OCD. This interpretation should nevertheless remain cautious, as the association between OCD clinical variables—including symptom severity and illness duration—and executive performance has varied across studies; clinical severity, comorbidity, affective symptoms, medication exposure, and task characteristics may all contribute to this heterogeneity [30]. From a phenomenological perspective, reactive obsessional presentations are characterized by externally triggered threat-related concerns and preventive behavioral responses; however, whether these qualitative features directly relate to executive performance remains speculative. Given the small subgroup sample and uncorrected analyses, these observed associations must be regarded strictly as exploratory and hypothesis-generating rather than as evidence of a distinct clinical–cognitive mechanism specific to reactive OCD.

Several nominal associations were observed in the autogenous group, including inverse associations between age at OCD onset and multiple RAVLT indices, namely initial recall, learning, recall after interference, short-delay recall, long-delay recall, and recognition, as well as a positive association with Stroop interference time. In addition, greater OCD symptom severity was nominally associated with lower long-delay recall and longer Stroop interference time, whereas higher depressive symptom severity was nominally associated with poorer recall after interference and short-delay recall. No significant associations were observed between the examined clinical variables and WCST Categories Completed (CC) or Perseverative Errors (PE) in the autogenous group. These nominal within-group associations should not be interpreted as evidence of a statistically distinct age-at-onset–memory or clinical–cognitive relationship in the autogenous group, as formal between-group comparisons of the corresponding correlation coefficients did not reveal significant differences. Although several nominal associations in this subgroup involved verbal learning and memory measures, this pattern does not establish that clinical characteristics are more closely related to memory than to executive-function measures in autogenous OCD. Although previous evidence regarding the relationship between age at OCD onset and cognitive performance remains limited and heterogeneous, one recent study reported an association between later age at onset and poorer performance across multiple neurocognitive domains in a cognitively impaired OCD subgroup [31]. These associations should nevertheless be interpreted cautiously, as age at onset may be related to current age and other unmeasured variables. Given the small subgroup sample sizes, multiple uncorrected comparisons, and the cross-sectional nature of these analyses, the findings should be considered exploratory and hypothesis-generating rather than evidence of a presentation-specific clinical–cognitive mechanism or a causal relationship.

When considered descriptively, the nominal within-group correlations may appear to differ across subgroups; however, such apparent differences should not be interpreted as demonstrated between-group differences. However, formal Fisher’s r-to-z comparisons did not reveal any statistically significant differences between the two groups for any of the examined correlation coefficients (Supplementary Table S1). Accordingly, the within-group correlation findings should be regarded as exploratory and hypothesis-generating, rather than as evidence of distinct clinical–cognitive mechanisms in autogenous and reactive OCD.

The present study has several strengths. First, patients were classified into strictly defined autogenous and reactive OCD groups based on clinical evaluation and the Y-BOCS Symptom Checklist, while mixed presentations were excluded. This approach minimized symptom overlap and enabled a more specific examination of potential cognitive differences related to obsessional symptom profiles. Second, the groups were comparable with respect to key demographic and clinical characteristics, including age, educational level, illness duration, and overall OCD symptom severity. Third, neuropsychological assessment was conducted under standardized conditions by the same investigator and covered complementary domains of executive functioning, interference control, verbal learning, and memory.

Several limitations should also be considered. First, the modest overall sample size and relatively small subgroup samples for the reactive and autogenous OCD groups may have reduced statistical power to detect subtle or small-to-moderate between-group neuropsychological differences. This limited sample size similarly constrains the stability of within-group correlation estimates, which may be sensitive to individual variability and should therefore be interpreted with caution. In addition, the cross-sectional design precludes causal inferences, and correlations that did not survive multiplicity correction should be interpreted cautiously as exploratory. The lack of a healthy control group prevents conclusions regarding the absolute level of cognitive impairment relative to the general population; accordingly, all findings must be interpreted strictly as relative differences or similarities between two distinct clinical OCD presentations rather than as evidence of absolute neurocognitive challenges. Furthermore, because all participants were receiving pharmacological treatment, medication-related effects on neuropsychological performance cannot be excluded. Although no significant between-group differences were observed in the recorded frequencies of SSRI, SNRI, TCA, or atypical antipsychotic use, detailed information regarding daily dosages, treatment duration, treatment response, cumulative medication exposure, and total anticholinergic burden was not systematically quantified. Consequently, differences in treatment intensity or pharmacological burden may have remained undetected. Residual medication-related confounding may therefore have influenced processing speed, attention, motivation, memory, or executive performance, and medication exposure could not be fully controlled for in the present analyses. In addition, although error counts and self-corrections on the Stroop Test were recorded, they demonstrated marked floor effects with minimal variance across participants; therefore, completion time on the fifth condition (color-word interference) was utilized as the primary index of inhibitory control. Importantly, the exclusion of participants with BDI scores ≥ 17 restricted the range of depressive symptom severity within the sample; this restriction of range may have attenuated the observed associations involving BDI scores, rendering these correlations relatively conservative. In addition, no formal estimate of premorbid or current intellectual functioning was obtained. Given that educational attainment was categorized into only three broad strata, potential residual confounding related to general cognitive ability cannot be excluded, which limits the interpretation of both between-group comparisons and correlational patterns. Accordingly, both the between-group comparative findings and the within-group correlational patterns reported here should be regarded as preliminary and hypothesis-generating, warranting replication in larger, adequately powered samples. Finally, although the pure-group design enhanced phenotypic specificity, the exclusion of mixed presentations may limit generalizability to the broader OCD population; moreover, traditional neuropsychological measures may not fully reflect everyday cognitive difficulties or metacognitive processes that may be particularly relevant to OCD.

Accordingly, all between-group comparative findings and within-group correlational patterns should be explicitly regarded as preliminary and hypothesis-generating, warranting replication in larger, adequately powered multicenter cohorts. Future research should examine autogenous and reactive obsessive symptom dimensions using both categorical and dimensional approaches in adequately powered samples. Longitudinal designs are needed to clarify whether changes in clinical characteristics and treatment status are associated with changes in cognitive performance over time. Future studies should also formally compare clinical–cognitive associations and use multivariable models to account for potential confounding factors. In addition to conventional neuropsychological measures, the assessment of memory confidence and related metacognitive processes may provide a more comprehensive account of cognitive heterogeneity in OCD [32]. Employing comprehensive psychometric tools to capture diverse metacognitive domains, as successfully utilized and conceptualized across psychiatric conditions [33], could help delineate whether domain-specific metacognitive beliefs or introspective accuracy differentiate obsessional presentations. Assessments of thought-control strategies, metacognitive beliefs, and real-world functional outcomes may further enhance the clinical relevance of future research.

## 5. Conclusions

In this exploratory cross-sectional study, patients with strictly classified autogenous and reactive obsessional presentations of OCD showed broadly comparable performance on conventional measures of executive functioning, attentional control, and verbal learning and memory, indicating that the autogenous–reactive distinction is not associated with a generalized neurocognitive divergence. The only nominally significant between-group difference was observed for RAVLT-6 performance, with better recall after interference in the autogenous group; however, because depressive symptom severity differed between groups and multiple neuropsychological outcomes were examined, this secondary observation should be interpreted cautiously and requires replication before firm conclusions can be drawn. Although exploratory within-group analyses descriptively suggested several clinical–cognitive associations, formal between-group comparisons of correlation coefficients revealed no statistically significant differences. Accordingly, these correlational patterns should be regarded strictly as hypothesis-generating rather than as evidence of distinct clinical–cognitive mechanisms in autogenous and reactive OCD, warranting further investigation in larger, adequately powered cohorts.

## Figures and Tables

**Table 3:** Pearson correlations between clinical variables and neuropsychological test performance in the reactive OCD group (*n* = 35).

Measure	Y-BOCS	BDI	Illness Duration	Age at OCD Onset
RAVLT-1 (Initial recall)	−0.211	−0.238	−0.007	−0.350 *
RAVLT-5 (Verbal learning)	−0.263	−0.227	0.058	−0.169
RAVLT-6 (Recall after interference)	−0.119	−0.044	−0.026	−0.394 *
RAVLT-7 (Short-delay recall)	−0.277	−0.277	0.149	−0.137
RAVLT-8 (Long-delay recall)	−0.307	−0.276	−0.036	0.048
Recognition performance	−0.156	−0.219	−0.114	−0.244
Stroop Test (Interference time)	0.469 **	0.287	0.403 *	0.373 *
WCST Categories Completed	−0.382 *	−0.331	−0.554 **^†^	−0.118
WCST Perseverative Errors	0.417 *	0.475 **	0.292	0.403 *
WCST Conceptual Level Responses	−0.252	−0.230	−0.334 *	0.018
**Associations Between Clinical Variables**				
Y-BOCS	-	0.725 **	0.380 *	0.057
BDI	0.725 **	-	0.477 **	0.145
Illness duration	0.380 *	0.477 **	-	−0.171
Age at OCD onset	0.057	0.145	−0.171	-

Values are Pearson correlation coefficients (r). OCD, obsessive–compulsive disorder; Y-BOCS, Yale–Brown Obsessive Compulsive Scale; BDI, Beck Depression Inventory; RAVLT, Rey Auditory Verbal Learning Test; WCST, Wisconsin Card Sorting Test. RAVLT-1 (Trial 1) reflects initial immediate recall of List A; RAVLT-5 (Trial 5) reflects recall after five learning trials of List A (verbal learning); RAVLT-6 (Trial 6) reflects recall of List A immediately after presentation and recall of the interference list (List B); RAVLT-7 reflects short-delay (20 min) recall of List A; RAVLT-8 reflects long-delay recall of List A; recognition performance reflects recognition discrimination for List A items. * *p* < 0.05, ** *p* < 0.01 (two-tailed, unadjusted). Benjamini–Hochberg false discovery rate (FDR) correction was applied across the 40 correlations between neuropsychological measures and clinical variables within this group; ^†^ denotes the single correlation (WCST Categories Completed–Illness Duration, q = 0.022) that remained significant after FDR correction. All other correlations, including those marked with * or **, did not survive FDR correction and should be interpreted as exploratory.

**Table 4:** Pearson correlations between clinical variables and neuropsychological test performance in the autogenous OCD group (*n* = 32).

Measure	Y-BOCS	BDI	Illness Duration	Age at OCD Onset
RAVLT-1 (Initial recall)	−0.329	−0.232	−0.199	−0.408 *
RAVLT-5 (Verbal learning)	−0.246	−0.234	−0.072	−0.391 *
RAVLT-6 (Recall after interference)	−0.311	−0.364 *	−0.119	−0.436 *
RAVLT-7 (Short-delay recall)	−0.321	−0.406 *	−0.250	−0.430 *
RAVLT-8 (Long-delay recall)	−0.351 *	−0.348	−0.296	−0.546 **^†^
Recognition performance	−0.281	−0.088	−0.328	−0.413 *
Stroop Test (Interference time)	0.362 *	0.269	0.120	0.596 **^†^
WCST Categories Completed	−0.224	−0.212	−0.184	−0.309
WCST Perseverative Errors	0.213	0.251	0.034	0.347
WCST Conceptual Level Responses	−0.132	−0.228	−0.370 *	−0.310
**Associations Between Clinical Variables**				
Y-BOCS	-	0.636 **	0.146	0.019
BDI	0.636 **	-	0.291	0.005
Illness duration	0.146	0.291	-	−0.201
Age at OCD onset	0.019	0.005	−0.201	-

Values are Pearson correlation coefficients (r). OCD, obsessive–compulsive disorder; Y-BOCS, Yale–Brown Obsessive Compulsive Scale; BDI, Beck Depression Inventory; RAVLT, Rey Auditory Verbal Learning Test; WCST, Wisconsin Card Sorting Test. RAVLT-1 (Trial 1) reflects initial immediate recall of List A; RAVLT-5 (Trial 5) reflects recall after five learning trials of List A (verbal learning); RAVLT-6 (Trial 6) reflects recall of List A immediately after presentation and recall of the interference list (List B); RAVLT-7 reflects short-delay (20 min) recall of List A; RAVLT-8 reflects long-delay recall of List A; recognition performance reflects recognition discrimination for List A items. * *p* < 0.05, ** *p* < 0.01 (two-tailed, unadjusted). Benjamini–Hochberg false discovery rate (FDR) correction was applied across the 40 correlations between neuropsychological measures and clinical variables within this group; ^†^ denotes correlations that remained significant after FDR correction (RAVLT-8–age at OCD onset, q = 0.025; Stroop interference time–age at OCD onset, q = 0.013). All other correlations, including those marked with * or **, did not survive FDR correction and should be interpreted as exploratory.

## Data Availability

The data presented in this study are available on request from the corresponding author due to privacy.

## Supplementary Materials

The following supporting information can be downloaded at: https://www.mdpi.com/article/10.3390/brainsci16090922/s1, Table S1: Fisher’s r-to-z comparisons of correlations between clinical variables and neuropsychological measures.
