# Supplementary material for: Comparison of Cognitive Functions in Patients with Autogenous and Reactive Obsessive–Compulsive Disorder
Source: Brain Sci. 2026 Aug 29;16(9):922. doi: 10.3390/brainsci16090922 (PMC13604620; doi:10.3390/brainsci16090922)
Supplement: Supplementary file 1 [file brainsci-16-00922-s001.zip › brainsci-4507615-supplementary.pdf]

Supplementary Table S1. Fisher's r-to-z comparisons of correlations between clinical variables and neuropsychological measures.

| Correlation pair | Reactive r (*n*) | Autogenous r (*n*) | Fisher z | p-value |
|---|---|---|---|---|
| Y-BOCS–RAVLT-8 | −0.307 (35) | −0.351 (32) | 0.193 | 0.847 |
| BDI–RAVLT-6 | −0.044 (35) | −0.364 (32) | 1.316 | 0.188 |
| BDI–RAVLT-7 | −0.277 (35) | −0.406 (32) | 0.571 | 0.568 |
| BDI–WCST perseverative errors | 0.475 (35) | 0.251 (32) | 1.014 | 0.310 |
| Illness duration–WCST categories completed | −0.554 (35) | −0.184 (32) | −1.708 | 0.088 |
| Age at OCD onset–RAVLT-1 | −0.350 (35) | −0.408 (32) | 0.264 | 0.792 |
| Age at OCD onset–RAVLT-6 | −0.394 (35) | −0.436 (32) | 0.198 | 0.843 |
| Age at OCD onset–Stroop fifth condition | 0.373 (35) | 0.596 (32) | −1.151 | 0.250 |
| Y-BOCS–Stroop fifth condition | 0.469 (35) | 0.362 (32) | 0.505 | 0.613 |
| Y-BOCS–WCST categories completed | −0.382 (35) | −0.224 (32) | −0.681 | 0.496 |
| Y-BOCS–WCST perseverative errors | 0.417 (35) | 0.213 (32) | 0.888 | 0.374 |

Note: Pearson correlation coefficients were calculated separately within each group. Fisher's r-to-z tests compared the independent correlation coefficients. None of the examined between-group differences was statistically significant at $p < 0.05$.
